# *catena*-Poly[[bis­(5-chloro-2-nitro­benzoato)copper(II)]-bis­(μ-5-chloro-2-nitro­benzoato)]

**DOI:** 10.1107/S1600536809001895

**Published:** 2009-01-23

**Authors:** Eng Khoon Lim, Siang Guan Teoh, Ibrahim Abdul Razak, Hoong-Kun Fun

**Affiliations:** a School of Chemical Sciences, Universiti Sains Malaysia, 11800 USM, Penang, Malaysia; b X-ray Crystallography Unit, School of Physics, Universiti Sains Malaysia, 11800 USM, Penang, Malaysia

## Abstract

In the title compound, [Cu_2_(C_7_H_3_ClNO_4_)_4_]_*n*_, the coordination geometry around each Cu^II^ ion is distorted square-pyramidal. The CuO_5_ coordination is formed by five O atoms from the carboxyl­ate groups of five 5-chloro-2-nitro­benzoate ligands. This coordination leads to the formation of centrosymmetric binuclear units which are edge-shared, forming a linear chain along the *a* axis, with the Cu^II^ ions alternately separated by 2.5891 (4) and 3.1763 (4) Å. The chains are inter­connected into a three-dimensional network by C—H⋯O inter­actions.

## Related literature

For general background, see: Balaraman *et al.* (2006 ▶); Tomoya *et al.* (2005 ▶). For bond-length data, see: Allen *et al.* (1987 ▶). For related structures, see: Kabbani *et al.* (2004 ▶); Stachová *et al.* (2004 ▶).
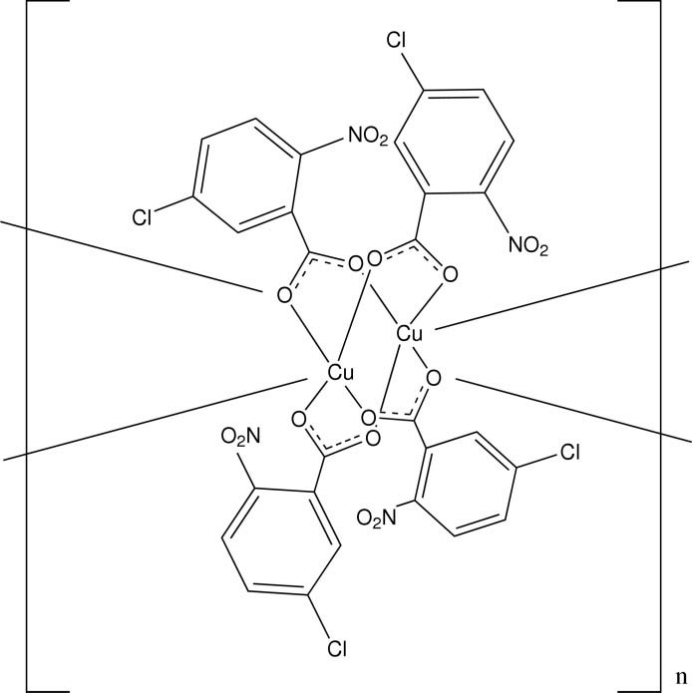


## Experimental

### 

#### Crystal data

[Cu_2_(C_7_H_3_ClNO_4_)_4_]
*M* *_r_* = 929.30
Triclinic, 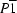

*a* = 5.0353 (1) Å
*b* = 11.8001 (3) Å
*c* = 13.8595 (3) Å
α = 84.539 (2)°
β = 85.553 (1)°
γ = 85.610 (2)°
*V* = 815.30 (3) Å^3^
*Z* = 1
Mo *K*α radiation
μ = 1.72 mm^−1^
*T* = 100.0 (1) K
0.47 × 0.21 × 0.08 mm

#### Data collection

Bruker APEXII CCD area-detector diffractometer
Absorption correction: multi-scan (*SADABS*; Bruker, 2005 ▶) *T*_min_ = 0.498, *T*_max_ = 0.875
11613 measured reflections
4656 independent reflections
3994 reflections with *I* > 2σ(*I*)
*R*_int_ = 0.034

#### Refinement

*R*[*F* ^2^ > 2σ(*F* ^2^)] = 0.035
*wR*(*F* ^2^) = 0.116
*S* = 1.10
4656 reflections
244 parameters
H-atom parameters constrained
Δρ_max_ = 0.72 e Å^−3^
Δρ_min_ = −1.04 e Å^−3^

### 

Data collection: *APEX2* (Bruker, 2005 ▶); cell refinement: *SAINT* (Bruker, 2005 ▶); data reduction: *SAINT*; program(s) used to solve structure: *SHELXTL* (Sheldrick, 2008 ▶); program(s) used to refine structure: *SHELXTL*; molecular graphics: *SHELXTL*; software used to prepare material for publication: *SHELXTL* and *PLATON* (Spek, 2003 ▶).

## Supplementary Material

Crystal structure: contains datablocks global, I. DOI: 10.1107/S1600536809001895/ci2748sup1.cif

Structure factors: contains datablocks I. DOI: 10.1107/S1600536809001895/ci2748Isup2.hkl

Additional supplementary materials:  crystallographic information; 3D view; checkCIF report

## Figures and Tables

**Table 1:** Selected bond lengths (Å)

Cu1—O5	1.942 (2)
Cu1—O6^i^	1.946 (2)
Cu1—O2^ii^	1.950 (2)
Cu1—O1^iii^	2.008 (2)
Cu1—O1	2.165 (2)

Symmetry codes: (i) 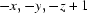; (ii) 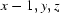; (iii) 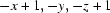.

**Table 2:** Hydrogen-bond geometry (Å, °)

*D*—H⋯*A*	*D*—H	H⋯*A*	*D*⋯*A*	*D*—H⋯*A*
C2—H2*A*⋯O4^iv^	0.93	2.44	3.254 (3)	146
C11—H11*A*⋯O8^v^	0.93	2.46	3.384 (3)	172
C14—H14*A*⋯O4^i^	0.93	2.54	3.417 (3)	156

Symmetry codes: (i) 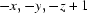; (iv) 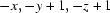; (v) 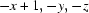.

**Fig. 1.**
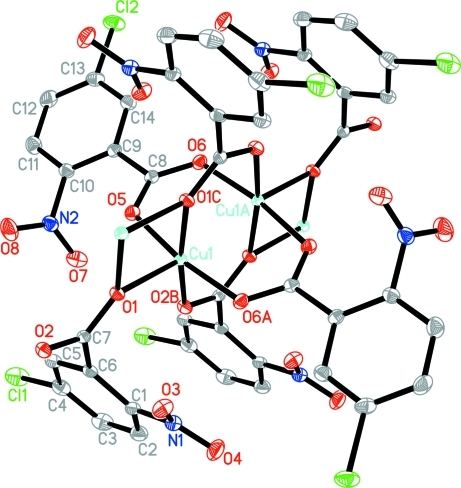
Part of the polymeric chain of the title compound, showing the coordination environment of the Cu atom and with displacement ellipsoids drawn at the 50% probability level. H-atoms are omitted for clarity. Symmetry codes: (A) -x, -y, 1-z; (B) x-1, y, z; (C) 1-x, -y, 1-z.

**Fig. 2.**
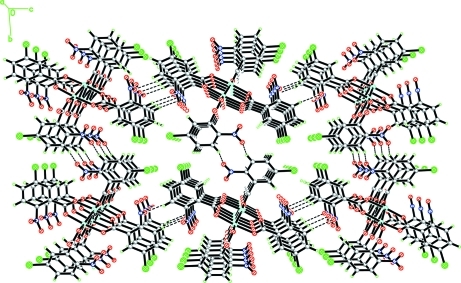
The crystal packing of the title compound, viewed down the a axis. Hydrogen bonds are shown as dashed lines.

## supplementary crystallographic information

### Comment

The ability to induce DNA cleavage in the presence of H_2_O_2_ and reductants by phenanthroline-based copper complexes such as [Cu(imda)(5,6-dmp)] (where 5,6-dmp is 5,6-dimethyl-1,10-phenanthroline) and [Cu(*N*,*N*'-dialkyl-1,10-phenanthroline -2,9-dimethanamine)] (Balaraman *et al.*, 2006; Tomoya *et al.*, 2005) have driven us to investigate the DNA cleavage ability of benzoic acid-based copper complexes. Several benzoic acid-based copper complexes have been prepared in our laboratory and their DNA cleavage abilities are further investigated. In this paper, we report the crystal structure of the title compound.

In the title compound, the coordination geometry around each Cu^II^ ion can be described as square-pyramidal, formed by five O atoms from the carboxylate groups of five 5-chloro-2-nitrobenzoate ligands. The basal plane positions are occupied by atoms O5, O6A, O2B and O1C with an average Cu—O bond length of 1.962 (2) Å. The apical position is occupied by atom O1 (Fig.1). The Cu1 atom is displaced away from the basal plane by 0.1689 (3) Å and the Cu—Cu(-*x*,-*y*,1 - *z*) separation is 2.5891 (4) Å. Similar CuO_5_ coordination were observed in related structures reported by Kabbani *et al.* (2004) and Stachová *et al.* (2004). The CuO_5_ square pyramids are edge-shared to form a linear polymeric chain along the *a* axis. In the chain, the Cu^II^ ions are alternately separated by 2.5891 (4) and 3.1763 (4) Å.

Bond lengths of the ligands have normal values (Allen *et al.*, 1987). The dihedral angle between nitro groups and the benzene rings are: C1–C6/N1/O3/O4 = 12.0 (3)° and C9–C14/N2/O7/O8 = 65.1 (3)°.

The polymeric chains are interconnected through C—H···O intramolecular interactions, forming a three-dimensional network (Table 2 and Fig. 2).

### Experimental

An ethanol solution (50 ml) of 5-chloro-2-nitrobenzoic acid (4.84 g, 0.024 mol) was added to a solution of copper(II) sulfate pentahydrate (3.00 g, 0.012 mol) in ethanol (50 ml) and the mixture was stirred and refluxed for 2 h. The resulting solution was filtered and left to cool down to room temperature. After a few days of slow evaporation, blue crystals suitable for X-ray analysis were collected.

### Refinement

All H atoms were positioned geometrically and refined using a riding model with C-H = 0.93 Å and U_iso_(H) =  1.2*U*_eq_(C).

### Crystal data

**Table:**

[Cu_2_(C_7_H_3_ClNO_4_)_4_]	*Z* = 1
*M**_r_* = 929.30	*F*(000) = 462
Triclinic, *P*1	*D*_x_ = 1.893 Mg m^−^^3^
Hall symbol: -P 1	Mo *K*α radiation, λ = 0.71073 Å
*a* = 5.0353 (1) Å	Cell parameters from 4198 reflections
*b* = 11.8001 (3) Å	θ = 2.4–33.5°
*c* = 13.8595 (3) Å	µ = 1.72 mm^−^^1^
α = 84.539 (2)°	*T* = 100 K
β = 85.553 (1)°	Plate, blue
γ = 85.610 (2)°	0.47 × 0.21 × 0.08 mm
*V* = 815.30 (3) Å^3^	

### Data collection

**Table:**

Bruker APEXII CCD area-detector diffractometer	4656 independent reflections
Radiation source: fine-focus sealed tube	3994 reflections with *I* > 2σ(*I*)
graphite	*R*_int_ = 0.034
φ and ω scans	θ_max_ = 30.0°, θ_min_ = 1.5°
Absorption correction: multi-scan (*SADABS*; Bruker, 2005)	*h* = −7→7
*T*_min_ = 0.498, *T*_max_ = 0.875	*k* = −16→16
11613 measured reflections	*l* = −19→19

### Refinement

**Table:**

Refinement on *F*^2^	Primary atom site location: structure-invariant direct methods
Least-squares matrix: full	Secondary atom site location: difference Fourier map
*R*[*F*^2^ > 2σ(*F*^2^)] = 0.035	Hydrogen site location: inferred from neighbouring sites
*wR*(*F*^2^) = 0.116	H-atom parameters constrained
*S* = 1.10	*w* = 1/[σ^2^(*F*_o_^2^) + (0.0656*P*)^2^ + 0.3146*P*] where *P* = (*F*_o_^2^ + 2*F*_c_^2^)/3
4656 reflections	(Δ/σ)_max_ = 0.001
244 parameters	Δρ_max_ = 0.72 e Å^−^^3^
0 restraints	Δρ_min_ = −1.04 e Å^−^^3^

### Special details

**Table:**

Experimental. The data was collected with the Oxford Cryosystem Cobra low-temperature attachment
Geometry. All e.s.d.'s (except the e.s.d. in the dihedral angle between two l.s. planes) are estimated using the full covariance matrix. The cell e.s.d.'s are taken into account individually in the estimation of e.s.d.'s in distances, angles and torsion angles; correlations between e.s.d.'s in cell parameters are only used when they are defined by crystal symmetry. An approximate (isotropic) treatment of cell e.s.d.'s is used for estimating e.s.d.'s involving l.s. planes.
Refinement. Refinement of *F*^2^ against ALL reflections. The weighted *R*-factor *wR* and goodness of fit *S* are based on *F*^2^, conventional *R*-factors *R* are based on *F*, with *F* set to zero for negative *F*^2^. The threshold expression of *F*^2^ > σ(*F*^2^) is used only for calculating *R*-factors(gt) *etc*. and is not relevant to the choice of reflections for refinement. *R*-factors based on *F*^2^ are statistically about twice as large as those based on *F*, and *R*- factors based on ALL data will be even larger.

### Fractional atomic coordinates and isotropic or equivalent isotropic displacement parameters (Å^2^)

**Table:**

	*x*	*y*	*z*	*U*_iso_*/*U*_eq_	
Cu1	0.21339 (5)	0.05196 (2)	0.471444 (19)	0.01008 (9)	
Cl1	0.39928 (16)	0.36470 (6)	0.09685 (5)	0.02730 (16)	
Cl2	−0.29870 (15)	−0.38843 (6)	0.13820 (5)	0.02494 (16)	
O1	0.6289 (3)	0.09053 (14)	0.45747 (12)	0.0112 (3)	
O2	1.0009 (3)	0.17993 (14)	0.40983 (12)	0.0130 (3)	
O3	0.6319 (4)	0.29482 (16)	0.56079 (13)	0.0190 (4)	
O4	0.2829 (4)	0.41394 (17)	0.56834 (15)	0.0242 (4)	
O5	0.2124 (3)	−0.02541 (15)	0.35393 (12)	0.0138 (3)	
O6	−0.1564 (3)	−0.11767 (15)	0.40442 (12)	0.0141 (3)	
O7	0.1420 (4)	0.10970 (17)	0.17610 (15)	0.0247 (4)	
O8	0.5268 (4)	0.0237 (2)	0.13998 (16)	0.0283 (5)	
N1	0.4497 (4)	0.35283 (18)	0.52330 (16)	0.0162 (4)	
N2	0.2865 (4)	0.02437 (19)	0.16027 (15)	0.0172 (4)	
C1	0.4307 (5)	0.3520 (2)	0.41832 (17)	0.0138 (4)	
C2	0.2609 (5)	0.4338 (2)	0.3721 (2)	0.0191 (5)	
H2A	0.1571	0.4864	0.4073	0.023*	
C3	0.2481 (5)	0.4361 (2)	0.2728 (2)	0.0215 (5)	
H3A	0.1339	0.4899	0.2403	0.026*	
C4	0.4063 (5)	0.3577 (2)	0.22185 (19)	0.0191 (5)	
C5	0.5750 (5)	0.2744 (2)	0.26839 (18)	0.0150 (4)	
H5A	0.6780	0.2219	0.2329	0.018*	
C6	0.5877 (4)	0.2705 (2)	0.36849 (18)	0.0128 (4)	
C7	0.7537 (4)	0.1749 (2)	0.41697 (16)	0.0116 (4)	
C8	0.0272 (4)	−0.0881 (2)	0.34205 (17)	0.0125 (4)	
C9	0.0247 (5)	−0.1317 (2)	0.24379 (17)	0.0131 (4)	
C10	0.1569 (5)	−0.0832 (2)	0.16019 (18)	0.0156 (5)	
C11	0.1618 (5)	−0.1279 (2)	0.07137 (19)	0.0209 (5)	
H11A	0.2577	−0.0948	0.0173	0.025*	
C12	0.0212 (5)	−0.2231 (2)	0.06434 (19)	0.0217 (5)	
H12A	0.0200	−0.2545	0.0053	0.026*	
C13	−0.1169 (5)	−0.2706 (2)	0.14635 (19)	0.0187 (5)	
C14	−0.1152 (5)	−0.2281 (2)	0.23593 (18)	0.0168 (5)	
H14A	−0.2060	−0.2632	0.2903	0.020*	

### Atomic displacement parameters (Å^2^)

**Table:**

	*U*^11^	*U*^22^	*U*^33^	*U*^12^	*U*^13^	*U*^23^
Cu1	0.00866 (14)	0.01013 (15)	0.01168 (15)	−0.00058 (9)	−0.00121 (9)	−0.00174 (10)
Cl1	0.0418 (4)	0.0235 (3)	0.0178 (3)	−0.0067 (3)	−0.0126 (3)	0.0037 (2)
Cl2	0.0375 (4)	0.0189 (3)	0.0210 (3)	−0.0106 (3)	−0.0089 (3)	−0.0029 (2)
O1	0.0094 (7)	0.0094 (8)	0.0142 (7)	0.0000 (6)	−0.0016 (6)	0.0014 (6)
O2	0.0087 (7)	0.0118 (8)	0.0181 (8)	0.0005 (6)	−0.0013 (6)	0.0012 (6)
O3	0.0224 (9)	0.0174 (9)	0.0175 (9)	0.0029 (7)	−0.0051 (7)	−0.0030 (7)
O4	0.0250 (10)	0.0228 (10)	0.0237 (10)	0.0053 (8)	0.0051 (8)	−0.0068 (8)
O5	0.0148 (8)	0.0154 (8)	0.0120 (7)	−0.0021 (6)	−0.0019 (6)	−0.0032 (6)
O6	0.0135 (7)	0.0151 (8)	0.0144 (8)	−0.0012 (6)	−0.0007 (6)	−0.0049 (6)
O7	0.0288 (10)	0.0179 (10)	0.0275 (10)	−0.0044 (8)	−0.0037 (8)	−0.0001 (8)
O8	0.0184 (9)	0.0366 (12)	0.0305 (11)	−0.0099 (8)	0.0025 (8)	−0.0026 (9)
N1	0.0169 (9)	0.0138 (10)	0.0181 (10)	−0.0024 (7)	0.0002 (8)	−0.0021 (8)
N2	0.0204 (10)	0.0178 (11)	0.0137 (9)	−0.0049 (8)	−0.0012 (8)	−0.0004 (8)
C1	0.0140 (10)	0.0113 (11)	0.0164 (11)	−0.0027 (8)	−0.0004 (8)	−0.0023 (8)
C2	0.0167 (11)	0.0139 (12)	0.0264 (13)	−0.0007 (9)	−0.0003 (9)	−0.0013 (10)
C3	0.0182 (11)	0.0162 (12)	0.0299 (14)	0.0012 (9)	−0.0099 (10)	0.0040 (10)
C4	0.0229 (12)	0.0191 (13)	0.0164 (11)	−0.0073 (10)	−0.0057 (9)	0.0014 (9)
C5	0.0178 (11)	0.0127 (11)	0.0149 (11)	−0.0032 (8)	−0.0024 (8)	−0.0005 (8)
C6	0.0100 (9)	0.0100 (10)	0.0185 (11)	−0.0021 (8)	−0.0015 (8)	0.0002 (8)
C7	0.0136 (10)	0.0113 (10)	0.0104 (9)	−0.0026 (8)	−0.0001 (8)	−0.0033 (8)
C8	0.0126 (10)	0.0114 (11)	0.0138 (10)	0.0001 (8)	−0.0018 (8)	−0.0022 (8)
C9	0.0132 (10)	0.0134 (11)	0.0132 (10)	−0.0001 (8)	−0.0025 (8)	−0.0032 (8)
C10	0.0149 (10)	0.0146 (11)	0.0176 (11)	−0.0027 (8)	−0.0012 (8)	−0.0010 (9)
C11	0.0232 (12)	0.0250 (14)	0.0145 (11)	−0.0040 (10)	−0.0002 (9)	−0.0012 (10)
C12	0.0273 (13)	0.0242 (14)	0.0151 (11)	−0.0021 (10)	−0.0033 (10)	−0.0078 (10)
C13	0.0222 (12)	0.0157 (12)	0.0197 (12)	−0.0031 (9)	−0.0063 (9)	−0.0033 (9)
C14	0.0196 (11)	0.0153 (12)	0.0155 (11)	−0.0029 (9)	−0.0016 (9)	0.0000 (9)

### Geometric parameters (Å, °)

**Table:**

Cu1—O5	1.942 (2)	C1—C2	1.384 (4)
Cu1—O6^i^	1.946 (2)	C1—C6	1.397 (3)
Cu1—O2^ii^	1.950 (2)	C2—C3	1.381 (4)
Cu1—O1^iii^	2.008 (2)	C2—H2A	0.93
Cu1—O1	2.165 (2)	C3—C4	1.383 (4)
Cu1—Cu1^i^	2.5891 (5)	C3—H3A	0.93
Cl1—C4	1.729 (3)	C4—C5	1.393 (4)
Cl2—C13	1.739 (3)	C5—C6	1.390 (3)
O1—C7	1.279 (3)	C5—H5A	0.93
O1—Cu1^iii^	2.0075 (17)	C6—C7	1.490 (3)
O2—C7	1.246 (3)	C8—C9	1.502 (3)
O2—Cu1^iv^	1.9501 (17)	C9—C10	1.388 (3)
O3—N1	1.221 (3)	C9—C14	1.400 (3)
O4—N1	1.236 (3)	C10—C11	1.382 (3)
O5—C8	1.263 (3)	C11—C12	1.388 (4)
O6—C8	1.262 (3)	C11—H11A	0.93
O6—Cu1^i^	1.9459 (16)	C12—C13	1.380 (4)
O7—N2	1.223 (3)	C12—H12A	0.93
O8—N2	1.220 (3)	C13—C14	1.383 (3)
N1—C1	1.466 (3)	C14—H14A	0.93
N2—C10	1.472 (3)		
			
O5—Cu1—O6^i^	170.11 (7)	C4—C3—H3A	120.3
O5—Cu1—O2^ii^	88.98 (7)	C3—C4—C5	121.7 (2)
O6^i^—Cu1—O2^ii^	90.41 (7)	C3—C4—Cl1	119.2 (2)
O5—Cu1—O1^iii^	90.80 (7)	C5—C4—Cl1	119.1 (2)
O6^i^—Cu1—O1^iii^	88.11 (7)	C6—C5—C4	119.3 (2)
O2^ii^—Cu1—O1^iii^	170.10 (6)	C6—C5—H5A	120.3
O5—Cu1—O1	97.86 (7)	C4—C5—H5A	120.3
O6^i^—Cu1—O1	91.67 (6)	C5—C6—C1	118.1 (2)
O2^ii^—Cu1—O1	108.91 (7)	C5—C6—C7	117.9 (2)
O1^iii^—Cu1—O1	80.92 (7)	C1—C6—C7	123.9 (2)
O5—Cu1—Cu1^i^	85.61 (5)	O2—C7—O1	125.4 (2)
O6^i^—Cu1—Cu1^i^	84.53 (5)	O2—C7—C6	118.2 (2)
O2^ii^—Cu1—Cu1^i^	90.98 (5)	O1—C7—C6	116.31 (19)
O1^iii^—Cu1—Cu1^i^	79.14 (5)	O6—C8—O5	126.5 (2)
O1—Cu1—Cu1^i^	159.80 (5)	O6—C8—C9	116.6 (2)
C7—O1—Cu1^iii^	127.17 (15)	O5—C8—C9	116.8 (2)
C7—O1—Cu1	133.75 (15)	C10—C9—C14	117.8 (2)
Cu1^iii^—O1—Cu1	99.07 (7)	C10—C9—C8	123.8 (2)
C7—O2—Cu1^iv^	117.23 (15)	C14—C9—C8	118.4 (2)
C8—O5—Cu1	120.91 (15)	C11—C10—C9	122.9 (2)
C8—O6—Cu1^i^	121.94 (15)	C11—C10—N2	115.9 (2)
O3—N1—O4	123.8 (2)	C9—C10—N2	121.1 (2)
O3—N1—C1	118.2 (2)	C10—C11—C12	118.8 (2)
O4—N1—C1	118.0 (2)	C10—C11—H11A	120.6
O8—N2—O7	124.6 (2)	C12—C11—H11A	120.6
O8—N2—C10	118.2 (2)	C13—C12—C11	119.0 (2)
O7—N2—C10	117.1 (2)	C13—C12—H12A	120.5
C2—C1—C6	122.5 (2)	C11—C12—H12A	120.5
C2—C1—N1	118.5 (2)	C12—C13—C14	122.2 (2)
C6—C1—N1	119.0 (2)	C12—C13—Cl2	119.5 (2)
C3—C2—C1	118.9 (2)	C14—C13—Cl2	118.3 (2)
C3—C2—H2A	120.6	C13—C14—C9	119.3 (2)
C1—C2—H2A	120.6	C13—C14—H14A	120.4
C2—C3—C4	119.5 (3)	C9—C14—H14A	120.4
C2—C3—H3A	120.3		
			
O5—Cu1—O1—C7	89.7 (2)	Cu1^iii^—O1—C7—O2	4.5 (3)
O6^i^—Cu1—O1—C7	−92.9 (2)	Cu1—O1—C7—O2	−174.59 (15)
O2^ii^—Cu1—O1—C7	−1.9 (2)	Cu1^iii^—O1—C7—C6	179.81 (14)
O1^iii^—Cu1—O1—C7	179.3 (2)	Cu1—O1—C7—C6	0.7 (3)
Cu1^i^—Cu1—O1—C7	−171.52 (14)	C5—C6—C7—O2	76.9 (3)
O5—Cu1—O1—Cu1^iii^	−89.55 (8)	C1—C6—C7—O2	−107.4 (3)
O6^i^—Cu1—O1—Cu1^iii^	87.82 (8)	C5—C6—C7—O1	−98.7 (2)
O2^ii^—Cu1—O1—Cu1^iii^	178.83 (6)	C1—C6—C7—O1	77.0 (3)
O1^iii^—Cu1—O1—Cu1^iii^	0.000 (2)	Cu1^i^—O6—C8—O5	8.8 (3)
Cu1^i^—Cu1—O1—Cu1^iii^	9.18 (17)	Cu1^i^—O6—C8—C9	−171.10 (15)
O2^ii^—Cu1—O5—C8	−88.27 (18)	Cu1—O5—C8—O6	−7.7 (3)
O1^iii^—Cu1—O5—C8	81.83 (18)	Cu1—O5—C8—C9	172.23 (15)
O1—Cu1—O5—C8	162.78 (18)	O6—C8—C9—C10	160.5 (2)
Cu1^i^—Cu1—O5—C8	2.79 (17)	O5—C8—C9—C10	−19.5 (4)
O3—N1—C1—C2	−167.1 (2)	O6—C8—C9—C14	−20.7 (3)
O4—N1—C1—C2	11.5 (3)	O5—C8—C9—C14	159.4 (2)
O3—N1—C1—C6	11.7 (3)	C14—C9—C10—C11	−1.9 (4)
O4—N1—C1—C6	−169.6 (2)	C8—C9—C10—C11	177.0 (2)
C6—C1—C2—C3	−0.8 (4)	C14—C9—C10—N2	174.1 (2)
N1—C1—C2—C3	177.9 (2)	C8—C9—C10—N2	−7.1 (4)
C1—C2—C3—C4	−0.6 (4)	O8—N2—C10—C11	−64.3 (3)
C2—C3—C4—C5	1.5 (4)	O7—N2—C10—C11	112.1 (3)
C2—C3—C4—Cl1	−177.4 (2)	O8—N2—C10—C9	119.4 (3)
C3—C4—C5—C6	−0.8 (4)	O7—N2—C10—C9	−64.1 (3)
Cl1—C4—C5—C6	178.02 (18)	C9—C10—C11—C12	2.3 (4)
C4—C5—C6—C1	−0.6 (3)	N2—C10—C11—C12	−173.9 (2)
C4—C5—C6—C7	175.3 (2)	C10—C11—C12—C13	−0.6 (4)
C2—C1—C6—C5	1.5 (3)	C11—C12—C13—C14	−1.5 (4)
N1—C1—C6—C5	−177.3 (2)	C11—C12—C13—Cl2	178.9 (2)
C2—C1—C6—C7	−174.2 (2)	C12—C13—C14—C9	1.8 (4)
N1—C1—C6—C7	7.0 (3)	Cl2—C13—C14—C9	−178.53 (19)
Cu1^iv^—O2—C7—O1	−3.4 (3)	C10—C9—C14—C13	−0.2 (4)
Cu1^iv^—O2—C7—C6	−178.60 (15)	C8—C9—C14—C13	−179.1 (2)

Symmetry codes: (i) −*x*, −*y*, −*z*+1; (ii) *x*−1, *y*, *z*; (iii) −*x*+1, −*y*, −*z*+1; (iv) *x*+1, *y*, *z*.

### Hydrogen-bond geometry (Å, °)

**Table:**

*D*—H···*A*	*D*—H	H···*A*	*D*···*A*	*D*—H···*A*
C2—H2A···O4^v^	0.93	2.44	3.254 (3)	146
C11—H11A···O8^vi^	0.93	2.46	3.384 (3)	172
C14—H14A···O4^i^	0.93	2.54	3.417 (3)	156

Symmetry codes: (v) −*x*, −*y*+1, −*z*+1; (vi) −*x*+1, −*y*, −*z*; (i) −*x*, −*y*, −*z*+1.
